# The accuracy of fluorine 18-labelled prostate-specific membrane antigen PET/CT and MRI for diagnosis of prostate cancer in PSA grey zone

**DOI:** 10.1038/s41416-024-02934-x

**Published:** 2024-12-19

**Authors:** Liang Luo, Ruiyan Wang, Lu Bai, Jin Shang, Xinyi Wang, Ruxi Chang, Weixuan Dong, Yang Li, Yan Li, Hua Liang, Hongjun Xie, Xiaoyi Duan

**Affiliations:** 1 PET/CT Center, The First Affiliated Hospital of Xi’ an Jiaotong University, Xi’ an, China; 2 Department of Radiology, The First Affiliated Hospital of Xi’an Jiaotong University, Xi’ an, China; 3 State Key Laboratory of Oncology in South China, Sun Yat-sen University Cancer Center, Guangzhou, China; 4 Department of Pathology, The First Affiliated Hospital of Xi’ an Jiaotong University, Xi’ an, China; 5 Department of Urology, The First Affiliated Hospital of Xi’ an Jiaotong University, Xi’ an, China

**Keywords:** Cancer imaging, Molecular imaging, Outcomes research

## Abstract

**Background:**

The diagnostic utility of prostate biopsy is limited for prostate cancer (PCa) in the prostate-specific antigen (PSA) grey zone. This study aims to evaluate the diagnostic performance of multiparametric magnetic resonance imaging (mpMRI) and prostate-specific membrane antigen positron emission tomography/computed tomography (PSMA PET/CT) for PSA grey zone PCa and clinically significant PCa (csPCa).

**Methods:**

A total of 82 patients with PSA levels ranging from 4 to 10 ng/mL who underwent ^18^F-PSMA-1007 PET/CT, mpMRI, and prostate biopsy were prospectively enrolled. For ^18^F-PSMA-1007 PET/CT and mpMRI in detecting PCa and csPCa, sensitivity, specificity, and area under the curve (AUC) were assessed using biopsy histology as the standard.

**Results:**

^18^F-PSMA-1007 PET/CT demonstrated better diagnostic performance for PCa than mpMRI (AUC 0.81 vs. 0.63, *P* = 0.02). 11.0% of patients with PI-RADS 3-5 had no PCa on biopsy, of whom 77.8% were correctly differentiated by ^18^F-PSMA-1007 PET/CT. Combined ^18^F-PSMA-1007 PET/CT + mpMRI improved sensitivity (92.5% vs. 73.6%) and negative predictive value (NPV, 78.9% vs. 53.3%) compared with mpMRI alone.

**Conclusions:**

^18^F-PSMA-1007 PET/CT outperformed mpMRI for detecting PCa in the grey zone level of PSA. ^18^F-PSMA-1007 PET/CT in combination with mpMRI has additional improvement in sensitivity and NPV for csPCa detection.

**Clinical Trial Registration:** NCT05958004, 2024-07.

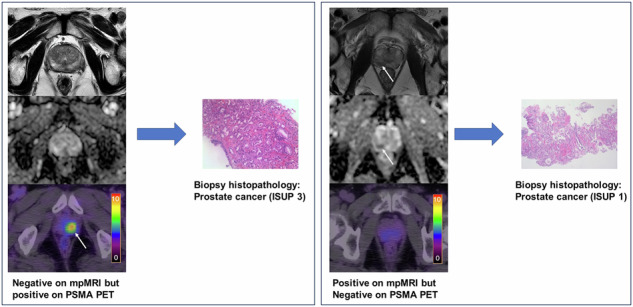

## Introduction

With the widespread serum prostate-specific antigen (PSA) tests, the number of prostate cancer (PCa) cases has risen rapidly in the last few decades [1]. However, an elevated PSA level could be caused by PCa and some non-malignant conditions, and the low specificity of the PSA screening may lead to potential overdiagnosis and overtreatment [2, 3]. Some patients will undergo unnecessary biopsies due to a false-positive PSA [4]. Moreover, the PCa detection rate of those with grey zone PSA levels ranging from 4 to 10 ng/mL on biopsy is between 16 and 39%, and the clinically significant prostate cancer (csPCa) detection rate is much lower [5]. Additionally, there is a significant probability of inaccurate samples and complications using biopsy, and it is difficult to discriminate between indolent and aggressive disorders [6, 7]. These obstacles prompted a growing interest in noninvasive and visual alternatives for early diagnosis of PCa and csPCa within PSA grey zone level, as well as reducing unneeded biopsies.

Multiparametric MRI (mpMRI) is an established imaging modality to obtain better sensitivity in the detection and localisation of csPCa and avoid biopsy [8, 9]. MRI-based prediction models that integrate clinical characteristics have demonstrated high predictive ability, and the Prostate Imaging Reporting and Data System (PI-RADS) score is one of the reliable variables for the diagnosis of PCa and csPCa in the PSA grey zone [10]. Nevertheless, mpMRI exhibits a low positive predictive value (PPV) for csPCa ranging from 34 to 68% [9, 11], and remains difficult to characterise PI-RADS 3 lesions, resulting in unnecessary biopsies. Prostate-specific membrane antigen (PSMA) PET/CT has been proven to outperform conventional imaging in detecting primary PCa [12–15], and it is an alternative and promising method to detect csPCa missed by mpMRI [16]. Studies have investigated the potential of PSMA PET/CT for the detection of intermediate- to high-risk intraprostatic tumour lesions [17–19], while the utility of detecting PSA grey zone PCa and csPCa in the pre-biopsy setting is uncertain. Therefore, this study evaluates the diagnostic accuracy of PSMA PET/CT in detecting PCa and csPCa in the PSA grey zone compared to mpMRI.

## Patients and methods

### Patients

Men with initial PSA levels of 4–10 ng/mL from June 2023 to March 2024 were consecutively enrolled in this prospective registered clinical trial (NCT 05958004). All recruited patients underwent prostate mpMRI, ^18^F-PSMA-1007 PET/CT, and prostate biopsy. The exclusion criteria included (1) previously known diagnosis of PCa; (2) history of other active malignancy; and (3) any absolute contra-indication to prostate mpMRI. This study was approved by the hospital ethics committee (No. KYLLSL-2023-248), and informed consent was obtained from all individual participants. Data from the research were collected and managed using Research Electronic Data Capture (REDCap) tools.

### mpMRI imaging acquisition and analysis

All candidates underwent prostate mpMRI performed in a 3.0-T scanner. The mpMRI protocol included T1-weighted images, T2-weighted images, diffusion-weighted images, and dynamic-contrast enhanced images. Abnormalities were reported by two experienced prostate radiologists who were unaware of clinical and pathological results according to PI-RADS version 2.1 [20]. Concordant conclusions regarding the abnormalities of mpMRI findings were required of the 2 readers. Positive mpMRI was defined as PI-RADS ≥ 3.

### PSMA PET/CT imaging acquisition

^18^F-PSMA-1007 was synthesised as described previously [21] and injected intravenously at a dose of 3.7 MBq/kg body weight. Patients underwent ^18^F-PSMA-1007 PET/CT scans 90 min after injection. PET attenuation was determined using low-dose CT scans from the head to the proximal thighs (pitch 0.8 mm, 60 mA, 140 kV [peak]) with 512 × 512 matrices. Whole-body PET scans were performed in three dimensions (emission time: 90 seconds per bed position, scanned at 7–10 beds) [21].

### PSMA PET/CT analysis and intra‑ and inter-observer variability analysis

Two qualified nuclear medicine physicians, blinded to the mpMRI and clinical outcomes, independently reviewed all PET/CT scans. A third read was performed to assess for discrepancies. The intraprostatic lesions were reviewed semi-quantitatively using the maximum standardised uptake value (SUVmax), based on the cutoff from our previous study [21] (PSMA-negative: SUVmax < 8.3, and PSMA-positive: SUVmax > 8.3), and also reported qualitatively based on their clinical experience using a four-point certainty scale (classifying “probably or definitely positive” as positive and “probably or definitely negative” as negative) [22]. ^18^F-PSMA-1007 PET/CT positivity was defined as either quantitatively positive and/or qualitatively positive.

Each observer re-assessed all cases at least two months following the first assessments to ascertain the intra-observer agreement in differentiating between PCa and non-PCa. The observers were blinded to their prior evaluations. The inter-observer variability of distinguishing between PCa and non-PCa was determined by comparing the consistency among observers.

### Prostate biopsy

An experienced urologist performed transperineal prostate systematic biopsies with a minimum of 12 cores. When possible, additional targeted biopsies were performed for patients with positive findings on mpMRI and/or ^18^F-PSMA-1007 PET/CT using a fusion biopsy system. The urologist will consider the key images to demonstrate the sites of positive lesions prior to biopsy. A maximum of three suspected lesions will be targeted, with one to three cores per lesion.

A specialised uropathologist assessed and interpreted each prostate biopsy in accordance with the guidelines established by the International Society of Urological Pathology (ISUP). The ISUP grade group of positive biopsies was documented. ISUP ≥ 2 indicates csPCa.

### Statistical analysis

The diagnostic value of mpMRI and ^18^F-PSMA-1007 PMSA PET/CT was evaluated using sensitivity, specificity, PPV, negative predictive value (NPV), and area under the curve (AUC). The performance of mpMRI (PI-RADS) and ^18^F-PSMA-1007 PMSA PET/CT (SUVmax) in differentiating between csPCa and non-csPCa lesions was assessed using a receiver operating characteristic (ROC) curve. To maximise sensitivity and specificity, the best cutoff value was determined using the Youden method. AUCs were compared using the DeLong test. Cohen’s weighted kappa method was used to estimate both intra- and inter-observer variability. Kappa values of 0 to 0.20 suggest weak agreement, 0.21 to 0.40 - fair agreement, 0.41 to 0.60 - moderate agreement, 0.61 to 0.80 - substantial agreement, and 0.81 to 1.0 - perfect agreement. Statistical analysis was carried out using MedCalc software (19.0.4). *P* < 0.05 was considered statistically significant.

## Results

### Participant characteristics

**Fig. 1:**
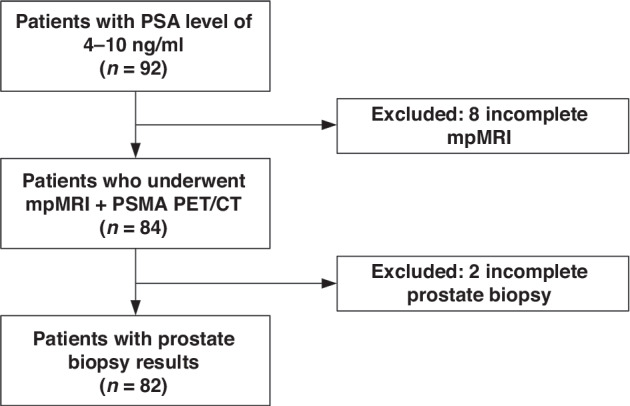
Flowchart.

From June 2023 to March 2024, 92 patients were prospectively recruited (Fig. 1). Of those, 2 patients without complete prostate biopsy and 8 patients without complete mpMRI were excluded due to the presence of metal implants or foreign bodies. The study comprised 82 participants with a median age of 67 years (IQR 62-72 years) who completed both imaging modalities and biopsy. Baseline characteristics are described in Table 1. The median time interval from mpMRI to ^18^F-PSMA-1007 PET/CT was 15 days (IQR 9–24 d). And the median PSA level was 7.9 ng/mL (IQR 6.5–9.4). Non-PCa were detected in 14 (17.1%) patients by biopsy histology, and PCa was detected in 68 (82.9%) patients, including 53 (64.6%) with csPCa (ISUP ≥ 2) and 15 (18.3%) with non-csPCa (ISUP 1).

**Table 1:** Baseline patient characteristics.

Characteristic	Median (interquartile range) or *n* (column %)
Age (y)	67 (62–72)
BMI (kg/m2)	24.3 (22.5–25.7)
PSA (ng/mL)	8.0 (6.5–9.4)
PSA density (ng/mL/cc)	0.19 (0.15–0.27)
Time from PSA to PSMA (d)	13 (8–24)
Time from MRI to biopsy (d)	6 (3–11)
Time from PSMA to biopsy (d)	7 (3–20)
PI-RADS	
1	6 (7.3)
2	5 (6.1)
3	19 (23.2)
4	42 (51.2)
5	10 (12.2)
PSMA SUVmax	9.5 (6.7–14.3)
ISUP grade group	
No cancer	14 (17.1)
1	15 (18.3)
2	19 (23.2)
3	13 (15.9)
4	12 (14.5)
5	9 (11.0)

*BMI* Body Mass Index, *PSA* prostate-specific antigen, *PI-RADS* Prostate Imaging Reporting and Data System, *PSMA* prostate-specific membrane antigen, *SUVmax* maximum standardised uptake value, *ISUP* International Society of Urological Pathology.

### Diagnostic performance of mpMRI and PSMA PET/CT

**Fig. 2:**
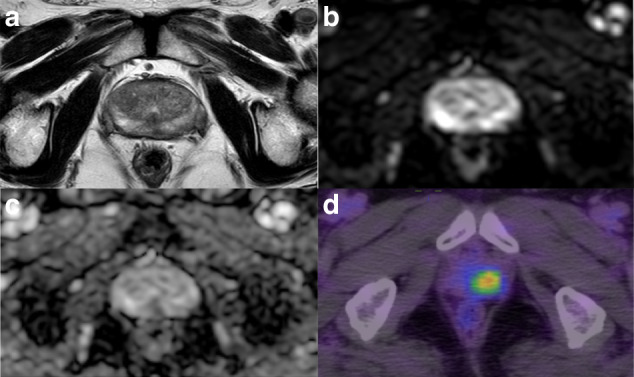
A positive lesion (SUVmax 14.3) on ^18^F-PSMA-1007 PET/CT was reported as PI-RADS 2 on mpMRI and confirmed to be prostate cancer on biopsy. (**a**) T2-weighted image, (**b**) diffusion-weighted image, (**c**) apparent diffusion coefficient map, and (**d**) ^18^F-PSMA-1007 PET/CT image.

**Fig. 3:**
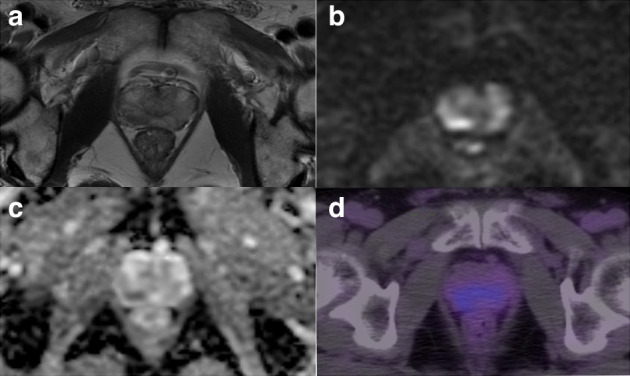
A PI-RADS 4 lesion on mpMRI was negative on ^18^F-PSMA-1007 PET/CT (SUVmax 4.5) and confirmed to be prostate cancer on biopsy. (**a**) T2-weighted image, (**b**) diffusion-weighted image, (**c**) apparent diffusion coefficient map, and (**d**) ^18^F-PSMA-1007 PET/CT images.

**Fig. 4:**
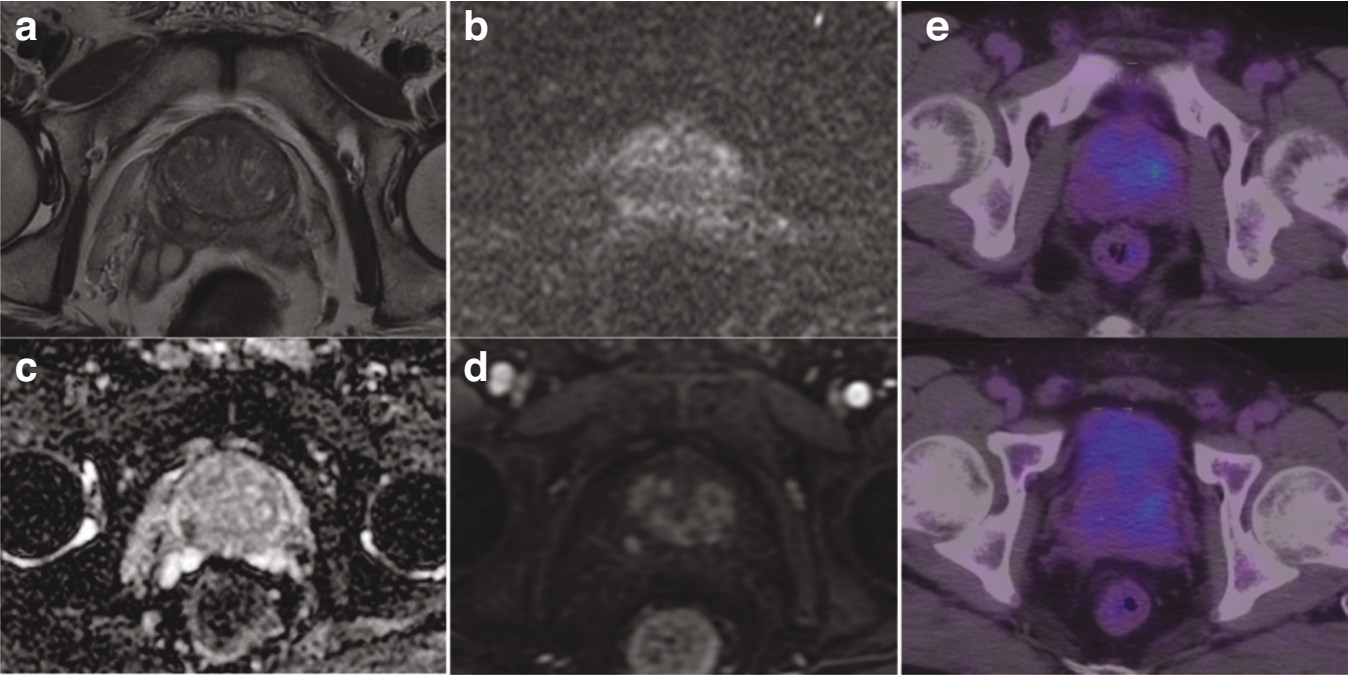
A PI-RADS 4 lesion on mpMRI was negative on ^18^F-PSMA-1007 PET/CT (SUVmax 6.1) and confirmed to be benign on biopsy. (**a**) T2-weighted image, (**b**) diffusion-weighted image, (**c**) apparent diffusion coefficient map, (**d**) dynamic-contrast enhanced image, and (**e**) ^18^F-PSMA-1007 PET/CT images.

Of the included 82 patients, 71 (86.8%) were classified as PCa based on mpMRI (PI-RADS of 3–5), 54 (65.9%) were considered positive on ^18^F-PSMA-1007 PET/CT, and 46 (56.1%) were positive on both modalities. Of the 68 biopsy-proven PCa patients, 6 (8.8%) were PSMA positive with PI-RADS 1-2 (Fig. 2), while 16 (23.5%) were PSMA negative with PI-RADS 3-5 (Fig. 3). Of the remaining 14 non-PCa patients, 9 (11.1%) were had PI-RADS ≥ 3, of whom 7 (77.8%) were PSMA negative (Fig. 4).

The proportion of men with csPCa and a PI-RADS of 1, 2, 3, 4, or 5 was 1/6, 3/5, 10/19, 31/42, and 8/10, respectively. Among men with positive ^18^F-PSMA-1007 PET/CT, 64.8% (35/54) had csPCa.

The diagnostic value of both imaging modalities for PCa with biopsy histology as the standard is shown in Table 2. The AUC of mpMRI alone was 0.63 (95% CI 0.52, 0.74), with sensitivity, specificity, PPV and NPV of 91.2% (95% CI 81.8%, 96.7%), 35.7% (95% CI 12.8%, 64.9%), 87.3% (95% CI 82.2%, 91.1%) and 45.5% (95% CI 22.8%, 70.2%), respectively. For ^18^F-PSMA-1007 PET/CT, the AUC was 0.81 (95% CI 0.71, 0.89), with sensitivity of 76.5% (95% CI 64.6%, 85.9%), specificity of 85.7% (95% CI 57.2%, 98.2%), PPV of 96.3% (95% CI 87.7%, 98.6%) and NPV of 42.9% (95% CI 31.7%, 54.7%). ^18^F-PSMA-1007 PET/CT showed greater performance than that of mpMRI (AUC 0.81 vs. 0.63, *P* = 0.02).

**Table 2:** Diagnostic performance of mpMRI and ^18^F-PSMA-1007 PET/CT for PCa in PSA grey zone level.

	mpMRI		PSMA PET/CT	
	Positive	Negative	Positive	Negative
PCa	62	6	52	16
Non-PCa	9	5	2	12
AUC	0.63 (0.52–0.74)		0.81 (0.71–0.89)	
Sensitivity (%)	91.2 (81.8–96.7)		76.5 (64.6–85.9)	
Specificity (%)	35.7 (12.8–64.9)		85.7 (57.2–98.2)	
PPV (%)	87.3 (82.2–91.1)		96.3 (87.7–98.6)	
NPV (%)	45.5 (22.8–70.2)		42.9 (31.7–54.7)	

*PCa* prostate cancer, *AUC* area under the curve, *PPV* positive predictive value, *NPV* negative predictive value.

For predicting csPCa, the AUC of mpMRI alone was 0.67 (95% CI 0.56, 0.77). The Youden-selected threshold of PI-RADS was 4. PI-RADS 4-5 vs. 1-3 had a specificity of 55.2% (95% CI 35.7%, 73.6%) and an NPV of 53.3% (95% CI 39.6%, 66.6%). The AUC of ^18^F-PSMA-1007 PET/CT alone was 0.71 (95% CI 0.60, 0.80), which is higher than that of mpMRI but not statistically significant (*P* = 0.58). A SUVmax cutoff of 9.3 showed an improvement in specificity (72.4% vs. 55.2%) and PPV (80.9% vs. 75.0%) compared with mpMRI, but the sensitivity (64.2% vs. 73.6%) and NPV (52.5% vs. 53.3%) were lower. Combined ^18^F-PSMA-1007 PET/CT + mpMRI showed a rise in NPV compared with mpMRI alone (78.9% vs. 53.3%, Table 3). The combination also improved sensitivity (92.5% vs. 73.6%), although specificity was slightly reduced (51.7% vs. 55.2%).

**Table 3:** Diagnostic performance of ^18^F-PSMA-1007 PET/CT, mpMRI, and PSMA + mpMRI for csPCa in PSA grey zone level.

	AUC	Sensitivity (%)	Specificity (%)	PPV (%)	NPV (%)
PSMA PET/CT	0.71 (0.60–0.80)	64.2 (49.8–76.9)	72.4 (52.8–87.3)	80.9 (69.5–88.8)	52.5 (42.0–62.8)
mpMRI	0.67 (0.56–0.77)	73.6 (59.7–84.7)	55.2 (35.7–73.6)	75.0 (66.0–82.3)	53.3 (39.6–66.6)
Combined PSMA + mpMRI	0.72 (0.61–0.81)	92.5 (81.8–97.9)	51.7 (32.5–70.6)	77.8 (70.4–83.7)	78.9 (57.8–91.1)

*csPCa* clinically significant prostate cancer, *AUC* area under the curve, *PPV* positive predictive value, *NPV* negative predictive value.

### Observer variability evaluation

All ^18^F-PSMA-1007 PET/CT scans were re-assessed, with results available for evaluation of inter- and intra-observer variability, respectively. In terms of predicting PCa, the kappa value of 0.64 (95% CI 0.46, 0.82) between the two observers suggested a substantial inter-observer agreement. Similarly, an average kappa value of 0.66 (95% CI 0.47, 0.85) indicated a substantial intra-observer agreement.

## Discussion

Diagnosis of PCa is critical for treatment decisions and prognosis outcomes, particularly in individuals within PSA grey zone levels. The low positive rate of biopsy for individuals with PCa and csPCa in PSA grey zone levels has resulted in underdiagnosis in some cases. A prospective clinical trial found that mpMRI could increase the rate of detecting csPCa on biopsy and decrease the number of biopsies performed [23]. Our study found that ^18^F-PSMA-1007 PET/CT had greater diagnostic accuracy than mpMRI for PCa and csPCa within PSA grey zone levels, although the difference is not significant in csPCa detection. Furthermore, combined ^18^F-PSMA-1007 PET/CT + mpMRI increased sensitivity and NPV for csPCa within PSA grey zone levels compared to mpMRI alone, potentially identifying individuals who could safely avoid biopsy.

Several studies have directly investigated the efficacy of ^18^F-PSMA-1007 PET/CT and mpMRI for diagnosing PCa and csPCa [22, 24]. A prospective single-arm trial [24] reported that mpMRI had greater diagnostic accuracy than ^68^Ga-PSMA-11 PET/CT for diagnosing PCa. The current study indicated that ^18^F-PSMA-1007 PET/CT had a similar lower sensitivity but higher specificity, probably due to the differences in PSA levels and radiotracers. The compelling benefits of ^18^F-PSMA-1007 PET/CT in this trial were in patients with positive mpMRI. 11.0% of patients with PI-RADS 3-5 had no PCa on biopsy, of whom 77.8% correctly differentiated by negative ^18^F-PSMA-1007 PET/CT. In addition, 56.1% of patients with positive mpMRI had positive ^18^F-PSMA-1007 PET/CT, of whom 95.8% had PCa on biopsy. Furthermore, ^18^F-PSMA-1007 PET/CT has certain advantages to mpMRI. First, PET/CT is regarded as a one-stop-shop scan [25], with scans ranging from the head to the proximal thighs, providing information on both primary and metastatic lesions. Second, mpMRI is not feasible for patients with metal implants, and ^18^F-PSMA-1007 PET/CT should be considered for this group of patients. Finally, the characteristics of PI-RADS 3 lesions in mpMRI are hard to determine. It has been demonstrated that PSMA PET/CT could be capable of stratifying PI-RADS 3 lesions on mpMRI [26, 27]. However, mpMRI will remain important in the primary setting because of its higher spatial resolution and the potential of false-positive PSMA PET [28].

^18^F-PSMA-1007 PET/CT and mpMRI both had a low NPV for csPCa within PSA grey zone levels. However, the synergistic impact of combining ^18^F-PSMA-1007 PET/CT and mpMRI was validated, and both sensitivity and NPV were better than ^18^F-PSMA-1007 PET/CT alone and mpMRI alone. Similarly, the PRIMARY study [22] found that combining ^68^Ga-PSMA-11 PET/CT with mpMRI increased sensitivity and NPV for the detection of csPCa. Given the high sensitivity and NPV of ^18^F-PSMA-1007 PET/CT + mpMRI in detecting csPCa, it may be safe to skip biopsy in patients who have negative ^18^F-PSMA-1007 PET/CT + mpMRI findings.

In this study, inter- and intra-observer agreement was substantial for detecting PCa. Similarly, Wondergem et al. [29] found a substantial interreader agreement (0.74) for ^18^F-PSMA-1007 PET/CT assessment of PCa. However, some variability was present in both inter-observer and intra-observer agreements, which may be related to the lack of appropriate reporting guidelines for intraprostatic lesions on ^18^F-PSMA-1007 PET/CT. Further improvement in clinical reporting criteria needs to be done to add the reliability of intraprostatic lesion detection across centres with varied equipment. In addition, integrating machine learning methods into ^18^F-PSMA-1007 PET/CT images could have the potential to increase diagnostic accuracy and consistency for detecting PSA grey zone PCa and csPCa.

There are certain limitations to our investigation. To begin, this trial used prostate biopsy histology rather than prostatectomy pathology to determine PCa and csPCa, which may have resulted in false-negative results or inaccurate Gleason score classification due to sampling error. To reduce the influence on our investigation, systematic biopsy in conjunction with mpMRI and/or ^18^F-PSMA-1007 PET/CT-based targeted biopsy was required for each case. Second, this is a single-centre study. Although all participants were enrolled and tested prospectively, the comparative analysis of ^18^F-PSMA-1007 PET/CT and mpMRI for PCa detection would have been influenced by the small sample of non-PCa. Third, 60% (3/5) of PI-RADS 2 lesions in this study were pathologically proven csPCa, a higher proportion compared with previous studies [22, 24], which could be related to the fact that there were only five PI-RADS 2 lesions in total. Therefore, larger sample sizes would be required to further validate the outcomes of the study. Fourth, our study did not carry out a lesion-based analysis, similar to the PRIMARY trial [22]. This is a trial to identify PCa and csPCa using ^18^F-PSMA-1007 PET/CT and mpMRI, and the findings may pave the way for future trials investigating the additional value of ^18^F-PSMA-1007 PET/CT over mpMRI in the detection of PSA grey zone PCa and csPCa.

In conclusion, ^18^F-PSMA-1007 PET/CT demonstrated greater diagnostic efficacy than mpMRI for the detection of PCa in the PSA grey zone, while not significantly higher when detecting csPCa in the PSA grey zone. Combined ^18^F-PSMA-1007 PET/CT + mpMRI has additional improvement in sensitivity and NPV. The inter-observer and intra-observer agreement of diagnosing PCa on ^18^F-PSMA-1007 PET/CT was substantial. ^18^F-PSMA-1007 PET/CT could be used for patients within PSA grey zone levels who are unable to undergo mpMRI.

## Data Availability

The dataset generated and analyzed during the current study is available from the corresponding author on reasonable request.
